# Life Course Tobacco Smoking and Risk of HPV-Negative Squamous Cell Carcinomas of Oral Cavity in Two Countries

**DOI:** 10.3389/froh.2022.844230

**Published:** 2022-03-30

**Authors:** Sreenath Madathil, Marie-Claude Rousseau, Doris Durán, Babatunde Y. Alli, Lawrence Joseph, Belinda Nicolau

**Affiliations:** ^1^Faculty of Dental Medicine and Oral Health Sciences, McGill University, Montréal, QC, Canada; ^2^Epidemiology and Biostatistics Unit, Institut Armand-Frappier, Institut National de la Recherche Scientifique (INRS), Laval, QC, Canada; ^3^Department of Epidemiology, Biostatistics and Occupational Health, McGill University, Montréal, QC, Canada; ^4^Facultad de Odontología, Instituto de Investigación en Ciencias Odontológicas, Universidad de Chile, Santiago, Chile

**Keywords:** life course epidemiology, Bayesian relevant life course exposure model, oral cancer, tobacco smoking, social and cultural factors

## Abstract

**Background:**

Tobacco smoking remains one of the major risk factors for oral cavity cancers (OCC), a subgroup of head and neck cancer (HNC) less attributed to human papillomavirus (HPV) infection. Although a strong dose-dependent association between tobacco smoking and OCC exists, several important questions on the age-dependent effects of this habit remain unanswered. We investigated which life course hypothesis best describes the association between tobacco smoking and HPV-negative (HPV^−*ve*^) OCC in Canada and India.

**Methods:**

We used data from the HeNCe Life study, a hospital-based case-control study conducted in Canada and India, using similar protocols. Cases were newly diagnosed subjects with primary squamous cell carcinomas of the head and neck region. Control subjects were patients with non-cancer selected from various outpatient clinics in a hospital located in the same catchment area as the cases and frequency-matched to cases according to age and sex. We collected information on an array of life course exposures using a structured questionnaire with the help of a life grid. Tobacco exposure (pack-years) during three life periods (≤ 30, 31−50, and >50 years of age) was calculated from the entire life course history of smoking. We used CDx brushes to collect oral exfoliated cells. Alpha HPV DNA detection and genotyping were performed for 36 HPV genotypes using the linear array. Participants who tested positive for HPV were excluded from the analysis. We used the Bayesian relevant life course exposure model (BRLM) to identify the life course hypothesis that best described the relationship between tobacco smoking and HPV^−*ve*^ OCC.

**Results:**

We show evidence for a late-life sensitive period (>50 years of age) for tobacco smoking in relation to the risk of HPV^−*ve*^ OCC in both Canada and India. An increase of 1 pack-year of tobacco smoking increased the risk of OCC by ~3% in both countries.

**Conclusion:**

Our findings from the Canadian and Indian data suggest that smoking tobacco after 50 years of age may carry a higher risk of developing oral cancer than earlier in life. Further studies are warranted to confirm the results.

## Introduction

Human papillomavirus (HPV) related head and neck cancers (HNCs) have been the focus of much attention in recent years due to a substantial increase in the incidence of these cancers [1, 2]. However, HPV-negative (HPV^−*ve*^) HNC, such as oral cavity cancers (OCC), remains a significant public health issue [2]. Moreover, there is evidence that the incidence of OCC is also rising in some populations [3], albeit less dramatically than HPV-positive (HPV^+ve^) HNC. A relatively recent international study reported that only 3.0–4.4% of OCC are attributable to oral HPV infection, with considerable heterogeneity in this attributable fraction across the globe with higher values in developed countries [4].

Tobacco and alcohol consumption account for more than 75% of the global incidence of OCC [5]. Several studies have shown a dose-dependent increase in OCC and a risk reduction after smoking cessation [5, 6]. However, the risk of OCC associated with different patterns of tobacco smoking over the life course is unknown. Moreover, while the risk attributable to tobacco smoking is similar in many countries, the absolute levels of tobacco consumption cannot explain the large differences in OCC incidence rates among countries. One potential explanation for these discrepancies concerns the relationship of smoking and socio-cultural factors (e.g., social and economic circumstances and cultural practices) with OCC development, which is not well-understood. Therefore, disentangling the varying effects of tobacco smoking on OCC etiology requires a comparison of these effects across populations with drastic socioeconomic and cultural differences and a consideration of smoking patterns over time.

Life course epidemiology [7] offers a framework to investigate the effects of time (duration) and timing of tobacco smoking on a later diagnosis of OCC. Three life course hypothetical models have been proposed: critical, sensitive, and accumulation hypotheses [8]. While the former two assume that exposures at specific life periods have greater importance in the disease process than other periods, the accumulation model suggests that all periods have equal importance with the effects accumulating over time.

No previous study has investigated which life course hypothetical models best describe the association between tobacco smoking and OCC. Moreover, life course studies on behavioral risk factors comparing results from different countries are rare. Cross-country studies can provide novel insights into the etiology of OCC and address important knowledge gaps concerning culturally specific pathways. If smoking patterns at different ages have different effects on the development of OCC, then such life-period-specific associations have high public health utility in designing effective primary prevention strategies. Moreover, given the lack of population-wide screening and OCC poor survival rates (the 5-year survival rate is ~50%), the main avenue for public health intervention is primary prevention [9].

This study is aimed to identify the life course hypothesis that best describes the association between tobacco smoking and HPV^−*ve*^ OCC in Canada and India.

## Materials and Methods

We use data from the Canadian and Indian sites of a hospital-based case-control study (HeNCe Life) established to investigate the risk factors of HNC. The study was conducted from 2005 to 2013 in major tertiary care centers in Montréal (Canada) and Kozhikode (India) using similar protocols. Details of the study design have been reported elsewhere [10, 11]. Briefly, cases (*n* = 460 in Canada; *n* = 350 in India) were consecutive patients with newly diagnosed, histologically confirmed squamous cell carcinomas of the head and neck region (oral cavity, pharynx, and larynx). Controls (*n* = 458 in Canada; *n* = 371 in India) were patients with non-cancer randomly selected from a defined list of diseases not associated with tobacco and alcohol from several outpatient clinics at the same hospitals as the cases. They were frequency-matched to the cases according to age (5-year groups) and sex.

One-on-one semi-structured interviews using a questionnaire and a life grid technique were conducted to collect information on several domains of exposures, such as demographics (e.g., age and sex), socioeconomic position (e.g., education, occupation, and income), and behavioral factors (e.g., tobacco and alcohol consumption and sexual behaviors).

Details of data collection procedures and life course exposure matrix creation are described in detail elsewhere [11, 12]. Briefly, a complete history of tobacco smoking was collected for each smoked tobacco product (cigarette, cigar, pipe, and bidi) as periods of a constant amount of consumption (units/day), brand and type (filtered, unfiltered, and hand-rolled). For each period, age at start and cessation were collected. To create comparable measures of tobacco smoking exposure between countries, we adopted a definition of standardized cigarette based on the tobacco content of different products: one pack of standardized cigarettes = 20 commercial cigarettes (filtered/unfiltered) = 4 hand-rolled cigarettes = 5 pipes = 4 cigars = 13.3 bidis [6, 13]. This information was used to calculate the intensity of tobacco smoking at each age of the participant (packs/day). Subsequently, the full life course exposure measures were cumulated into three life periods based on the distribution of the data: ≤ 30, 31−50, and >50 years of age.

We used oral rinse and brush biopsy protocols to collect oral exfoliated cells. HPV DNA detection and genotyping were performed using the Linear Array assay [11]. Probes for 36 HPV genotypes were used, and infection with HPV-52 was confirmed using PCR [11]. Participants with a valid β-globin detection who tested negative for all 36 genotypes were classified as HPV^−*ve*^. The results of the HPV analysis are reported elsewhere [11, 12]. In summary, none of the cases or controls tested positive for HPV-DNA at the India site of the study [12], while HPV was highly prevalent among Canadians [11].

### Statistical Analysis

**Figure 1:**
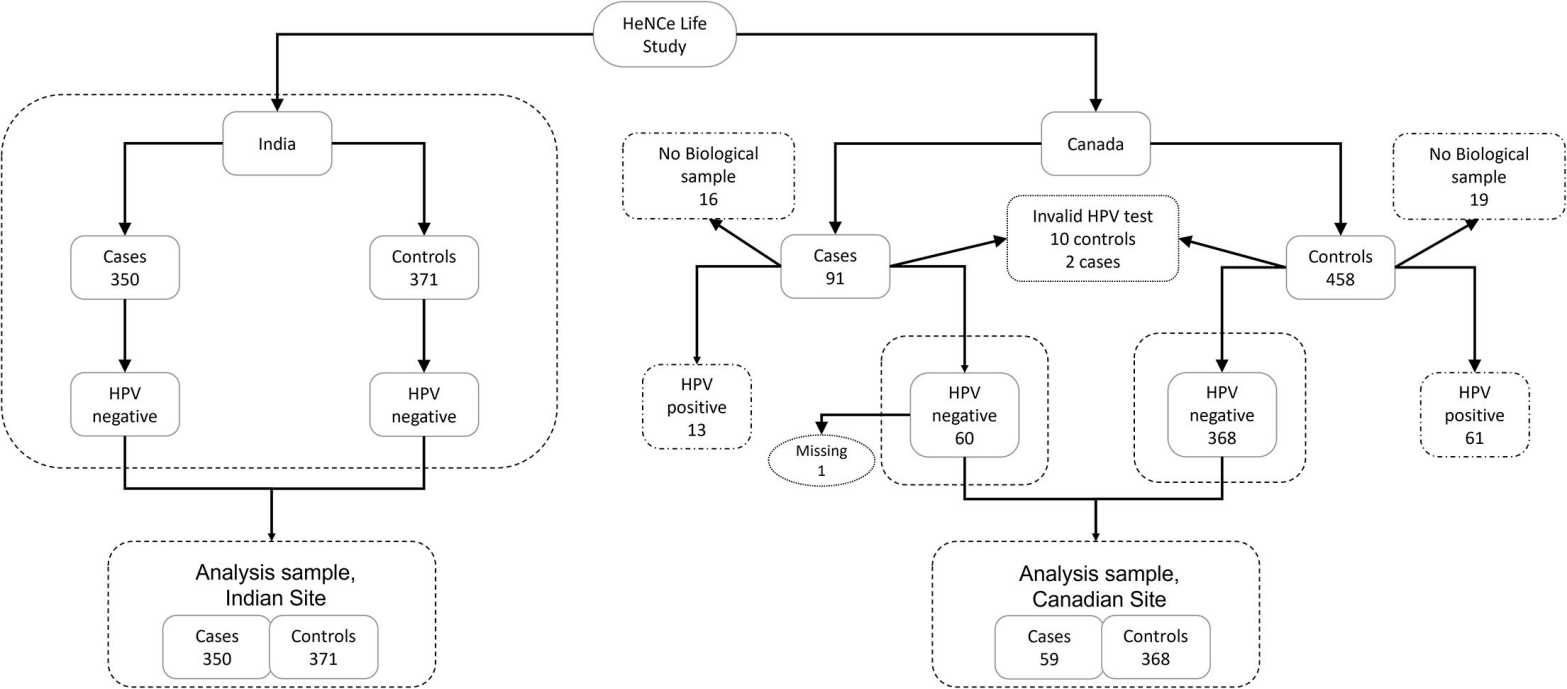
Study flowchart for the HeNCe Life Study in India and Canada.

To fulfill our objective, we restricted our analytical sample to HPV^−*ve*^ oral squamous cell carcinomas only. One OCC case at the Canadian site was excluded due to missing information on tobacco smoking. Therefore, our final analytical sample included all OCC cases (*n* = 350) and controls (*n* = 371) from the Indian site, and the majority of OCC cases (59 [64.8%]) and controls (368 [80.3%]) from the Canadian site (Figure 1).

Descriptive statistics were used to explore the data. We used the Bayesian relevant life course exposure model (BRLM) with the logistic likelihood function to investigate the life course hypotheses [14]. The technique proceeds by first assuming a weight for the amount (pack-years) of smoked tobacco exposure that occurred during each life period. The weights are further assumed to take values between 0 and 1 and sum to 1. The sum of reweighted exposures is termed as the “relevant life course exposure.” Thus, the weights denote the contribution of exposure that occurred during a period to the overall lifetime exposure that is relevant for the development of OCC.

The effect of 1 pack-year increase in this relevant life course exposure is termed as the “overall lifetime effect.” This formulation assumes that the effect of cumulative exposure depends on the periods during which the individual smoked.

In BRLM, the values for weights and the overall lifetime effect are estimated from the data. The life course hypothesis that best explains the relationship between tobacco smoking and OCC can then be inferred by comparing the values of estimated weights. For example, an accumulation hypothesis will result in the same value for all weights, whereas a critical period hypothesis will be inferred when the corresponding weight takes a value close to 1 [14].

Analyses were done separately for each country. The model for Canadian data was adjusted for age, sex, years of education, and lifetime liter-years of ethanol, whereas the analysis of Indian data was adjusted for age, sex, material deprivation index, lifetime liter-years of ethanol, and lifetime chew-years of betel quid. Additionally, 37 (5.1%) participants in the Indian dataset had missing values for the material deprivation index variable. To fully propagate the uncertainty around the missing values to the overall inference on life course hypotheses, we included an imputation model for the variable. Since this variable represents the number of items possessed out of 34 indicators of deprivation [15], we used a binomial regression model for imputation (as shown in Supplementary Materials for details).

**Figure 2:**
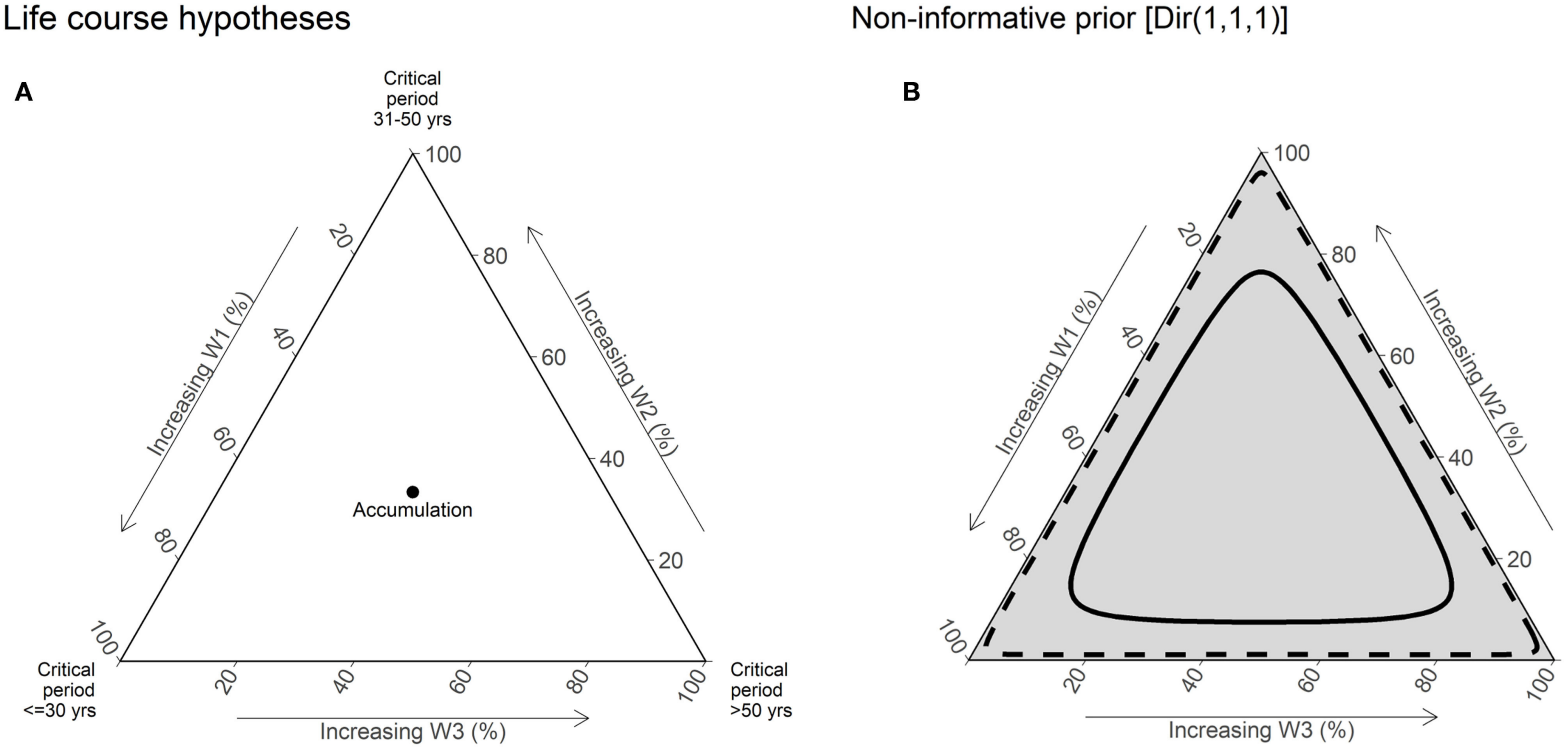
Illustration of life course hypotheses and non-informative prior distribution [Dir (1,1,1)]. **(A)** This ternary plot is used to visualize the joint distribution of weights for three periods. Vertices of the plot represent the extreme values of weights ([1,0,1] or [0,1,0] or [0,0,1]), and thus critical period hypotheses. The central point of the plot where the weights have equal value [0.333, 0.333, and 0.333] represents the accumulation hypotheses. The edges of the plot are labeled in % scale, which increases toward the corresponding vertices. **(B)** The gray shading of the plot represents the relative density of the prior joint distribution at specific point s in the plot. In other words, the prior belief on the value of weights. The solid line represents the 50% credible interval (CrI), and the dashed line represents the 95% CrI of the joint distribution. Under a non-informative prior distribution, all points in the plot are assumed to have equal probability and hence are shaded equally.

Prior distributions for weights can be used to incorporate previous knowledge about life course hypotheses. We used a non-informative prior distribution for weights [Dirichlet (1,1,1)], which is equivalent to assume that all life course hypotheses are equally probable (Figure 2).

Tobacco smoking has a dose-dependent positive association with OCC [16]. Hence, we constrained the overall lifetime effect parameter to be non-negative using the half Student's *t*-distribution [Student_t(3,0,2.5)T(0, )]. Posterior distributions were visualized as ternary plots and summarized using median and 95% credible intervals (95% *CrI*). Continuous covariates were scaled to *z*-scores to facilitate convergence. Weakly informative priors were used for all parameters [Student_t(3,0,5) for intercepts, Student_t(3,0,2.5), and Normal (0,2.5) for slopes].

## Results

Table 1 presents the distribution of socio-demographic and behavioral risk factors for HPV^−*ve*^ OCC cases and controls from both study sites. The proportions of ever smokers were slightly higher among OCC cases (Canada = 74.6% and India = 46.0%) than controls (Canada = 71.2% and India = 42.3%) in both countries. The lifetime cumulative exposure among smokers, calculated in pack-years, was higher among cases than controls in both countries.

**Table 1:** The distribution of selected socio-demographic and behavioral risk factors among human papillomavirus negative (HPV^−*negative*^) oral cavity cancer cases and controls in the HeNCe Life study: Canada and India sites.

	**Canada (*****n*** **=** **440)**		**India (*****n*** **=** **721)**	
	**Cases** **(*n* = 59)**	**Controls** **(*n* = 368)**	**Cases** **(*n* = 350)**	**Controls** **(*n* = 371)**
**Sex**				
Female	30 (50.8)	125 (34.0)	154 (44.0)	167 (45.0)
Male	29 (49.2)	243 (66.0)	196 (56.0)	204 (55.0)
**Age** (Mean ± SD)	62.41 ± 14.36	61.15 ± 11.14	60.77 ± 11.2	60.53 ± 11.7
**Tobacco smoking**
Never smoker	15 (25.4)	106 (28.8)	189 (54.0)	214 (57.7)
Ever smoker	44 (74.6)	262 (71.2)	161 (46.0)	157 (42.3)
**Pack-years** (Mean ± SD)^*^	43.92 ± 24.01	32.98 ± 32.91	34.88 ± 32.67	28.77 ± 34.09
Life stages: smoking pack-years^*^
≤ 30 years of age	14.58 ± 8.09	13.80 ± 12.19	10.06 ± 11.55	8.57 ± 11.79
31–50 years of age	19.91 ± 11.86	14.67 ± 17.71	18.16 ± 17.72	15.02 ± 18.67
>50 years of age	9.42 ± 10.10	4.51 ± 9.91	6.67 ± 9.27	5.18 ± 9.07
**Alcohol consumption**
Never drinker	10 (16.9)	66 (17.9)	253 (72.3)	306 (82.5)
Ever drinker	49 (83.1)	302 (82.1)	97 (27.7)	65 (17.5)
Ethanol liter-years (Mean ± SD)^#^	2.33 ± 4.55	1.03 ± 1.85	2.73 ± 5.17	1.03 ± 2.35
**Betel quid chewing**
Never chewer			97 (27.7)	306 (82.5)
Ever chewer			253 (72.3)	65 (17.5)
**Chew-years** (Mean ± SD)^**¥**^			267.46 ± 192.58	203.25 ± 207.08

* *Among ever smokers only*;

# *among ever drinkers only*;

¥ *among ever chewers only*.

**Figure 3:**
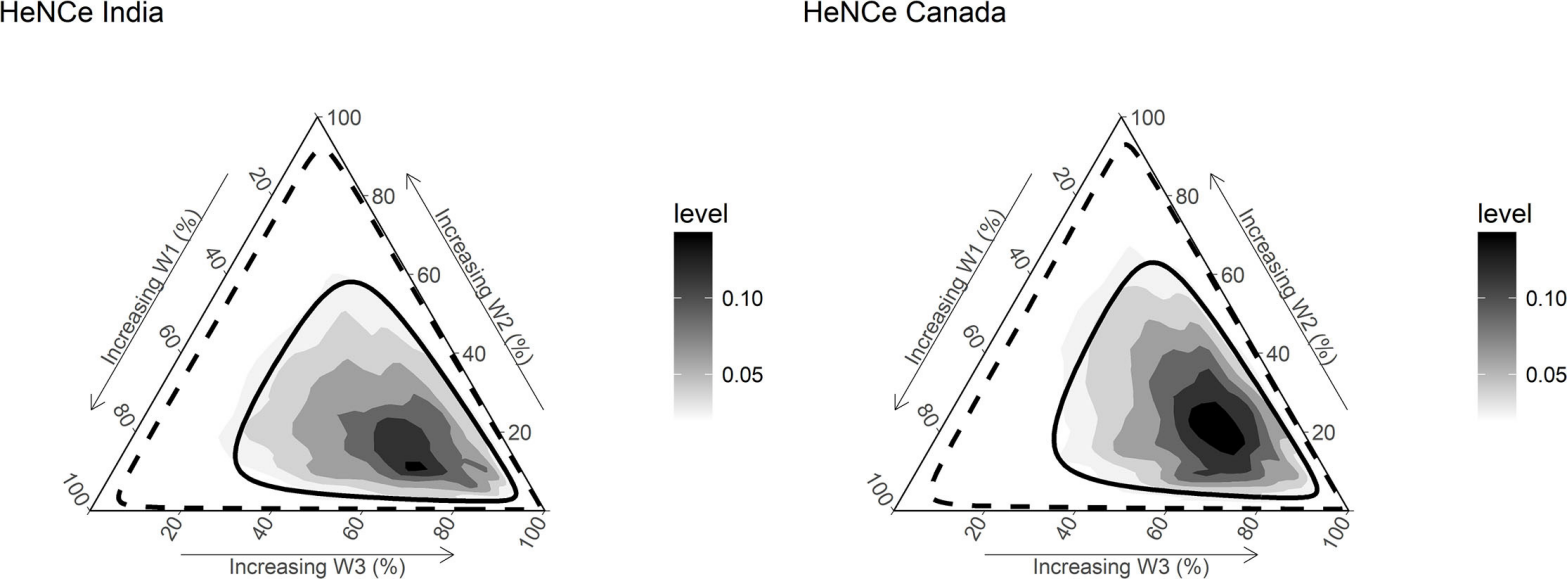
Densities and credible limits of posterior distributions of weights for exposure to tobacco smoking during three time periods [≤ 30 years (w1), 31–50 years (w2), and >50 years (w3)] with regards to risk for developing head and neck cancer (HNC), among human papillomavirus negative (HPV^−*ve*^) participants. Darker areas represent higher densities. The solid line represents the 50% credible limit, and the dashed line represents the 95% credible limit. The posterior distributions have relatively higher density toward the right lower vertices of the ternary plot, suggesting that the weights combinations with a higher value for the weight corresponding to the life period >50 years have a higher probability compared with others. In turn, the findings support a late-life (>50 years) sensitive period hypothesis.

Figure 3 presents the posterior distributions of weights for exposures at different periods estimated from the model. Interestingly, results from both Canada and India showed evidence for a sensitive period later in life (>50 years period), as illustrated by the higher density found in the lower right portion of the triangle.

The numerical summaries of posterior distributions of weights are reported in Table 2. Although the 95% *CrI*s overlap considerably, exposure after 50 years of age contributed to more than half of the life course exposure to tobacco smoking relevant for developing HPV^−*ve*^ OCC in the two countries. The mean weight estimated for tobacco smoking after 50 years of age was ~2 times that for other two periods.

**Table 2:** The relative importance of smoked tobacco exposure at different life periods for the risk of developing HPV^−*negative*^ oral cavity cancer (OCC), HeNCe Life study Canadian and Indian sites.

**Life period** **(age, in years)**	**Canada (%)**			**India (%)**		
	**Mean**	**Median**	**95% *CrI***	**Mean**	**Median**	**95% *CrI***
≤ 30	20.0	15.5	0.04–54.30	23.5	18.0	0.01–64.80
31–50	24.1	20.8	0.00–65.00	20.7	14.6	0.00–62.20
>50	54.9	57.8	9.14–94.20	55.8	59.8	6.82–96.40

The posterior probability that the third period had a higher weight compared with the second and first periods was 68.3 and 68.5% in the Canadian and Indian datasets, respectively. The prior probability of this hypothesis (based on a non-informative prior) was only ~33%.

Among ever smokers, each pack-year increase in the relevant life course exposure to smoked tobacco increased the risk of HPV^−*ve*^ OCC, by 2 and 3% in Canada (odds ratio [OR] = 1.02; 95% *CrI* = 1.00–1.05) and India (*OR* = 1.03; 95% *CrI* = 1.00–1.06), respectively. However, this effect depends on the period in which the increase happened. For example, smoking 1 pack of standardized cigarettes per day for 1 year (1 pack-year) after 50 years had the same effect on risk of OCC as smoking 2 packs per day for 1 year (2 pack-years) before 30 years or between 30 and 50 years.

## Discussion

There is strong evidence for the effect of tobacco use on the etiology of OCC, and a dose-dependent relationship between the habit and OCC risk is well-established. However, it is unknown whether early or late smoking is more strongly associated with an increased risk of OCC. We adopted a life course approach to address this knowledge gap. We investigated the effect of the timing of tobacco smoking on the risk of HPV^−*ve*^ OCC using a novel Bayesian life course exposure model. Data from Canada and India supported a late adulthood (>50 years of age) sensitive period model. Furthermore, there was ~68% posterior probability for this life course hypothesis.

Although a late adulthood sensitive period may seem counter intuitive, it is important to consider that a life course effect may be a combination of several factors, such as the latency of exposure, dose-dependent effects, and age-related vulnerability. The literature on tobacco smoking and lung cancer based on multistage carcinogenesis theory suggests that smoking may affect both the early and late stages of carcinogenesis [17–20]. A late adulthood sensitive period model may indicate that smoking has a stronger effect on the late stage of carcinogenesis in oral cancers.

A late adulthood sensitive period could also be due to a large threshold effect. Although previous studies have not reported a threshold effect of tobacco smoking in OCC, a non-linear dose-response relationship has been reported with risk leveling off at high cumulative exposures [21]. However, most of the previous studies have used lifetime cumulative pack-years of exposure, contributing to measurement error by also considering exposures occurring after the biological initiation of the disease. If indeed, there is a large threshold effect, that is the minimum exposure needed to start affecting carcinogenesis is high, a longer duration of exposure may be needed to achieve the cumulative threshold. This may result in a late life sensitive period if most of the participants are reaching the threshold after 50 years. Additionally, literature from lung cancer shows some evidence for an increase in risk due to the early initiation of smoking [22].

The above two potential explanations for a late life sensitive period, based on latency and threshold effects, need to be further investigated using longitudinal and mechanistic studies. Moreover, recent work from our group on the latency of tobacco consumption and the risk of HNC, including OCC, reported that the effect of tobacco on HNC risk was higher in patients with HVP^−*ve*^. Additionally, smoking later in life had a stronger association with cancer in those without HPV compared with those who had an HPV infection [23], agreeing with our current findings.

Socio-cultural factors may influence life course effects [24]. However, the observation of a late sensitive period in both Canada and India, countries presenting considerable differences in culture and burden of OCC, may be an indication that the reasons may be mechanistic rather than socio-cultural.

### Limitations

Our study is not devoid of limitations. Even after an *a priori* non-negative constraint on the overall lifetime effect of smoking, only weak evidence was observed with the lower limit of the 95% *CrI* interval very close to 1. This may reflect the low sample size combined with wide prior distributions on parameters. Moreover, in India, betel quid chewing is a stronger OCC risk factor compared with tobacco smoking, with approximately half of the national incidence attributed to the former. Furthermore, very few (<2%) of the women in the Indian dataset ever smoked tobacco, but the majority of them were users of betel quid. Unfortunately, due to low sample sizes, analyses could not be stratified by sex of participants.

Although alcohol consumption and betel quid chewing were measured throughout the life course of the participants, we considered only cumulative lifetime exposure of these confounders, which may have led to some residual confounding. We previously identified an early life (≤ 20 years) sensitive period for betel quid chewing using the same dataset (HeNCe Life study India) [14]. In the current analysis, we only considered cumulative exposure to betel quid, which might have influenced our results. However, the same life period was also identified as sensitive in the Canadian dataset, in which there was no exposure to betel quid chewing. In other words, obtaining very similar results from another country (Canada), where the confounding structure is potentially different, supports the notion that the late life sensitive period for smoking is not solely an artifact due to the existence of an early life sensitive period for betel quid chewing.

Three life periods (≤ 30, 31–50, and >50 years) were chosen in the current analysis for two main pragmatic reasons: (i) to obtain an approximately equal duration of exposure in these periods among ever smokers, on average (9–10 years); (ii) ease of interpretation. The choice of cut-off points to define these periods may have implications on the results. Further simulation studies are needed to fully characterize these implications.

Approximately 5% of participants at the Indian site had missing values for the material deprivation index variable. We accounted for these missing values by using an imputation model as part of the life course analysis model. This strategy allows for the propagation of the uncertainty in the missing values to the process of identification of life course hypotheses. Biological samples were not available for 16 (17.5%) OCC cases and 19 (4.1%) controls in the Canadian dataset. Additionally, among participants with biological samples, invalid results were obtained for 2 (2.6%) OCC cases and 10 (2.2%) controls. Furthermore, one participant at the Canadian site had missing information on smoking. Unfortunately, we were not able to adopt an imputation model for these data because: (i) there was not enough information in the dataset to effectively estimate the probability of a valid HPV result; (ii) analysis was restricted to HPV^−*ve*^ participants. However, because the reasons for not collecting a biological sample for a participant were mostly administrative/logistic at the beginning of recruitment, we believe that the resulting bias would be minimal.

The analytical technique we adopted did not allow adjusting for time-dependent confounding or interaction between tobacco smoking at two different periods. However, assessing mediation and interaction effects are the next steps in the scientific enquiry [25, 26] and future studies may use advanced causal inference techniques to achieve this [27, 28].

Collecting tobacco smoking exposures retrospectively relies heavily on the accuracy of recall. We used a life grid technique to aid the participants to recall specific details of their smoking history [29, 30]. Although the technique has been shown to improve the accuracy even after 50 years in some situations, the possibility of measurement error cannot be ruled out. However, because cases and controls followed the same protocol in both countries, the measurement error is likely to be non-differential and may have led to an underestimation of the effects.

Ours is the first study investigating which life course hypothesis best explains the relationship between tobacco smoking and HPV^−*ve*^ OCC in two countries. Considering the limitations of our study, it is more conservative to conclude that our results are only suggestive of a late life sensitive period.

## Conclusion

Results from our analyses, applying a new Bayesian relevant life course model, and contrasting results from two countries, are suggestive of a sensitive period for tobacco smoking after 50 years of age on the risk of OCC. Future studies are needed to better understand the phenomena.

## Data Availability Statement

The raw data supporting the conclusions of this article will be made available by the authors upon reasonable request for collaborative work, without undue reservation.

## Ethics Statement

The studies involving human participants were reviewed and approved by Research Ethics Office (IRB) of the Faculty of Medicine and Health Sciences, McGill University. The patients/participants provided their written informed consent to participate in this study.

## Author Contributions

SM and BN contributed to the conception, data collection, data analysis and interpretation, drafted, and critically revised the manuscript. M-CR and LJ contributed to conception, design, data analysis and interpretation, and critically revised the manuscript. BA and DD assisted in the interpretation of results and critically revised the manuscript. All authors have approved the final version of the manuscript.

## Funding

This HeNCe Life study was funded by CIHR grant numbers: MOP-69062, MOP-201009, DCA-122700, and the Ministere du Developpement economique, de l'Innovation et de l'Exportation du Quebec. BN holds a Canada Research Chair in life course oral epidemiology. SM was a recipient of a Career Award from the Fonds de Recherche du Québec—Santé (FRQ-S). BA was a recipient of doctoral training fellowship from FRQ-S. DD received financial support for her doctoral studies from Réseau de Recherche en Santé Buccodentaire et Osseuse for the year 2021–2022, and is a Kuok Fellow from the Rossy Cancer Network 2022–25.

## Conflict of Interest

The authors declare that the research was conducted in the absence of any commercial or financial relationships that could be construed as a potential conflict of interest.

## Publisher's Note

All claims expressed in this article are solely those of the authors and do not necessarily represent those of their affiliated organizations, or those of the publisher, the editors and the reviewers. Any product that may be evaluated in this article, or claim that may be made by its manufacturer, is not guaranteed or endorsed by the publisher.
