# Supplementary material for: Life Course Tobacco Smoking and Risk of HPV-Negative Squamous Cell Carcinomas of Oral Cavity in Two Countries
Source: Front Oral Health. 2022 Mar 30;3:844230. doi: 10.3389/froh.2022.844230 (PMC9005739; doi:10.3389/froh.2022.844230)
Supplement: Supplementary file 1 [file Data_Sheet_1.PDF]

Supplementary Material

Manuscript- Life course tobacco smoking and risk of HPV-negative oral cancers in two countries

Model for imputing missing values in material deprivation index in HeNCe Life study Indian dataset

Material deprivation index was created as a sum of binary variables for 34 different amenities. Hence, the variable can be assumed to be a binomial variable with a trial size of 34. Age, sex,indicator variable for the interviewer, education(high vs low), area living(urban vs rural), year of birth, years lived at the place of birth, Lifetime maximum income, number of meals per day, cumulative exposure tobacco smoking(pack-years), cumulative exposure of alcohol(liters of ethanol), and cumulative exposure to betel quid chewing (chew-years) were used as predictors in the imputation model. Weekly informative priors were used for coefficients of all predictors. Code to fit BRLM with the imputation model is as follows:

```
model{
  #---variables-------
  # N = total sample size
  # ncon = number of confounders
  # ncon2 = number of predictors for imputation model
  # X = matrix of exposure meausures in three periods
  # y = case-control status
  # conMat = matrix of confounders
  # mdi = material deprivation index
  # conMat2 = matrix of standardized covariates for imputation model (including intercept)
  #---------------------

#Likelihoods

  for(n in 1:N){

  #Analysis model
      logit(xb[n]) = alpha + delta*(X[n,1:pos[n]] %*% w[1:pos[n]]) +
              (conMat[n,1:ncon] %*% beta[1:ncon]) + beta[ncon+1]*mdi[n]

      y[n] ~ dbern(xb[n])

  #Model for imputation (Binomial model)
      logit(xb2[n]) = conMat2[n,1:ncon2] %*% gamma[1:ncon2]
      mdi[n] ~ dbin(xb2[n],34)
  }

#Priors for fixed effect

  alpha ~ dt(0,0.04,3)

  delta ~ dt(0,0.16,3)T(0,)

  beta[ncon+1] ~ dt(0,0.16,3)

  for(i in 1:ncon){
    beta[i] ~ dnorm(0,1)
  }

#Priors for imputation model (normal(0,1) & normal(0,5))
  gamma[1] ~ dnorm(0,0.04)
```

```
  for(i in 2:ncon2){
    gamma[i] ~ dnorm(0,1)
  }


#First age cohort has only two periods of exposures

  w[1:t] ~ ddirch(p_alpha[1:t])

#Calculating ORs
  OR = exp(delta)

#Hypos
  hyp = (w[3] > w[1]) && (w[3] > w[2])
}
```

Detailed output of model fit using runjags package

Table 2 - Detailed output of mcmc sampling of posterior distributions. HeNCe Life Study- India

```
Calculating summary statistics...
Calculating the Gelman-Rubin statistic for 28 variables....

JAGS model summary statistics from 40000 samples (chains = 4; adapt+burnin = 5000):
```

	Lower95	Median	Upper95	Mean	SD	Mode
alpha	1.4744	2.0448	2.6579	2.0471	0.30327	2.0443
gamma[1]	-0.18049	-0.11965	-0.053727	-0.11954	0.032587	-0.11795
gamma[2]	-0.31979	-0.16832	-0.030351	-0.17008	0.074285	-0.16506
gamma[3]	-0.4717	-0.39614	-0.32174	-0.39631	0.038582	-0.39321
gamma[4]	0.55954	0.62356	0.68319	0.62338	0.031332	0.62392
gamma[5]	0.24428	0.3134	0.3823	0.31336	0.035422	0.3138
gamma[6]	0.1135	0.17919	0.2445	0.17926	0.033354	0.17836
gamma[7]	-0.092866	0.052148	0.19471	0.050463	0.074061	0.057567
gamma[8]	0.13333	0.22362	0.3179	0.22386	0.047033	0.22421
gamma[9]	-0.01967	0.0090214	0.039361	0.0090441	0.015111	0.0083982
gamma[10]	0.27622	0.32259	0.36776	0.32262	0.02332	0.3207
gamma[11]	0.064369	0.092828	0.1224	0.09286	0.014779	0.093562
gamma[12]	-0.056033	-0.022743	0.011284	-0.022791	0.017144	-0.021959
gamma[13]	-0.073084	-0.043921	-0.016321	-0.043902	0.014517	-0.0436
gamma[14]	-0.20455	-0.17426	-0.14304	-0.17423	0.015778	-0.17419
beta[1]	-0.83201	-0.61119	-0.40221	-0.61298	0.10904	-0.60655
beta[2]	-0.47336	0.087537	0.63289	0.088983	0.28289	0.094236
beta[3]	-0.56372	-0.14158	0.26207	-0.1417	0.21138	-0.13947
beta[4]	0.94685	1.2315	1.5209	1.2348	0.14699	1.216
beta[5]	0.096865	0.39386	0.73822	0.40628	0.16681	0.37525
beta[6]	-0.47371	0.099607	0.66583	0.097548	0.29203	0.10935
beta[7]	-0.15297	-0.12109	-0.089739	-0.12114	0.016236	-0.12151
delta	1.267e-06	0.016798	0.047664	0.019687	0.014631	0.0080459
w[1]	0.00011299	0.1801	0.64809	0.23545	0.2005	0.080124
w[2]	0.000042392	0.14563	0.62175	0.20669	0.19445	0.061787
w[3]	0.068184	0.5975	0.96417	0.55786	0.25901	0.72646
hyp	0	1	1	0.6865	0.46392	1
OR	1	1.0169	1.0488	1.02	0.015021	1.0081

	MCerr	MC%ofSD	SSeff	AC.10	psrf
alpha	0.0081986	2.7	1368	0.50939	1.0002
gamma[1]	0.00063243	1.9	2655	0.2722	1.0004
gamma[2]	0.0034903	4.7	453	0.794	1.0045
gamma[3]	0.00069072	1.8	3120	0.22533	1.0001
gamma[4]	0.00032516	1	9285	0.019147	1.0005
gamma[5]	0.00038596	1.1	8423	0.049188	1.0004
gamma[6]	0.00037437	1.1	7937	0.037575	1.0012
gamma[7]	0.0034645	4.7	457	0.79389	1.0048
gamma[8]	0.00036288	0.8	16798	0.0032384	1.0003
gamma[9]	0.0001327	0.9	12967	0.031992	1.0001
gamma[10]	0.00028194	1.2	6841	0.077657	1.0001
gamma[11]	0.00011273	0.8	17189	0.015249	1.0002
gamma[12]	0.00019222	1.1	7954	0.052341	1.0001
gamma[13]	0.00010347	0.7	19685	0.0055852	1.0001

	MCerr	MC%ofSD	SSeff	AC.10	psrf
gamma[14]	0.00012473	0.8	16001	0.0086958	1
beta[1]	0.0013531	1.2	6493	0.095067	1.0001
beta[2]	0.0050283	1.8	3165	0.19994	1.0005
beta[3]	0.0022271	1.1	9008	0.033991	1.0003
beta[4]	0.0011628	0.8	15980	0.0040908	1.0004
beta[5]	0.0012532	0.8	17718	0.0015027	1.0003
beta[6]	0.0053912	1.8	2934	0.20644	1.0008
beta[7]	0.00043745	2.7	1378	0.50012	1.0003
delta	0.00023021	1.6	4039	0.15627	1.0003
w[1]	0.0038477	1.9	2715	0.27324	1.0014
w[2]	0.0039202	2	2460	0.3128	1.0018
w[3]	0.0058038	2.2	1992	0.37892	1.0035
hyp	0.0087178	1.9	2832	0.24855	1.0018
OR	0.00023621	1.6	4044	0.1559	1.0005

Total time taken: 7 minutes

Table 3 - Detailed output of mcmc sampling of posterior distributions.HeNCe Life study- Canada

Calculating summary statistics...
Calculating the Gelman-Rubin statistic for 12 variables....

JAGS model summary statistics from 40000 samples (chains = 4; adapt+burnin = 5000):

	Lower95	Median	Upper95	Mean	SD	Mode
betas[1]	-2.1391	-1.5328	-0.91116	-1.5401	0.3149	-1.5304
betas[2]	-0.38505	-0.064706	0.24352	-0.066364	0.15997	-0.066026
betas[3]	-1.5569	-0.9424	-0.32146	-0.94295	0.31444	-0.93049
betas[4]	-0.86783	-0.52947	-0.19918	-0.53128	0.17073	-0.52309
betas[5]	0.098744	0.32573	0.57154	0.32757	0.12099	0.31849
betas[6]	-0.94502	-0.187	0.53481	-0.18634	0.3758	-0.19775
delta	0.000026504	0.025576	0.054043	0.026834	0.015374	0.023511
w[1]	2.7038e-06	0.16466	0.59197	0.21202	0.18129	0.075761
w[2]	0.000012397	0.18409	0.66669	0.24201	0.20724	0.081435
w[3]	0.040274	0.58459	0.93649	0.54597	0.25316	0.68622
hyp	0	1	1	0.68315	0.46525	1
OR	1	1.0259	1.0555	1.0273	0.015851	1.0238

	MCerr	MC%ofSD	SSeff	AC.10	psrf
betas[1]	0.0052039	1.7	3662	0.17143	1.0008
betas[2]	0.0012299	0.8	16919	0.0012777	1.0002
betas[3]	0.0035077	1.1	8036	0.015277	1
betas[4]	0.0013649	0.8	15647	0.0010351	1
betas[5]	0.00092868	0.8	16973	0.015196	1
betas[6]	0.006443	1.7	3402	0.18618	1.0005
delta	0.00021789	1.4	4978	0.11067	1.001
w[1]	0.0035376	2	2626	0.26969	1.0021
w[2]	0.0042702	2.1	2355	0.31151	1.0048
w[3]	0.0055643	2.2	2070	0.36418	1.0062
hyp	0.0087057	1.9	2856	0.26271	1.0032
OR	0.00022457	1.4	4982	0.11032	1.001

Total time taken: 1.2 minutes
